# Equestrian-associated injuries of the hand: a retrospective analysis of injury mechanisms and patterns

**DOI:** 10.1007/s00402-024-05586-x

**Published:** 2024-10-17

**Authors:** Benedikt Ritter, Nadjib Dastagir, Martynas Tamulevicius, Florian Bucher, Doha Obed, Peter M. Vogt, Khaled Dastagir

**Affiliations:** Department of Plastic, Aesthetic, Hand and Reconstructive Surgery, Hannover Medical School, Carl-Neuberg-Str. 1, D-30625 Hannover, Germany

**Keywords:** Equestrian-associated injuries, Horse riding, Hand trauma, Hand injuries, Bridle

## Abstract

**Introduction:**

Hand injuries are frequently caused by sports and are associated with long periods of inability to work and high economic health costs. After ball sports and cycling, the most common cause of hand injuries is horseback riding. Therefore, measures should be taken to prevent these risks and increase safety in sports, however data characterizing equestrian sports-associated injuries are limited.

**Materials and Methods:**

A retrospective monocentric cohort study was performed including 39 patients (mean age: 35 ± 2.6 (range 9–65) years, female 89.7% who sustained an injury to the hand while practicing equestrian sports during 2016–2021 and presented to our hand surgery center. Data analysis was performed to characterize the trauma mechanism and injury patterns by evaluating the clinical information system and conducting telephone interviews.

**Results:**

Overall, 53.8% of the injuries occurred while leading the horse owing to traction by bridles or a lead rope on the fingers, whereas only 33.3% were caused by a fall and 12.8% by a bite injury. The majority (87.2%) of cases were injuries to the phalanges (metacarpus: 7.7%; carpus: 5.1%). Fractures were present in 51.3% of cases. The most serious injuries included avulsion amputations in 23.1% of patients (10.3% subtotal; 12.8% total amputation).

**Conclusions:**

Equestrian-associated injuries occur more frequently during horse handling than riding, resulting in severe avulsion amputations due to traction of the lunge or bridle, requiring complex microsurgical treatment. We recommend that appropriate protective gloves are worn for prevention of hand injuries. Additionally, the use of self-opening panic hooks with overload protection can prevent excessive traction.

**Level of evidence:**

III

## Introduction

Horseback riding is one of the oldest sports practiced by humans. To date, the oldest humans identified as horsemen are five Yamnaya individuals from kurgans in Romania, Bulgaria, and Hungary, dating from 3021 BCE to 2501 BCE, with alterations in bone morphology and marked pathologies associated with horseback riding [1]. In modern times, with industrialization and the spread of automobiles, horseback riding has become less important as a means of transportation; however, at the same time, it has developed into a sports and recreational activity. The transformation of the horse from a farm animal to a “sporting machine,” and the physiological and behavioral characteristics of the animal make equestrian sport high-risk. Depending on the breed, racehorses can reach a height of up to 2.20 m, a weight of up to 1200 kg, and speeds of up to 65 km/h. Moreover, the force of a hoof strike can reach 1.8 times the horse’s body weight. Despite the large number of active riders and horses being unpredictable, there is little data on injuries and accident mechanisms in mass riding [2].

Hand injuries are frequently caused by sports and are associated with long periods of inability to work and high economic costs [3]. After cycling and playing soccer, horseback riding is the third most common cause of hand injuries. Injuries caused by horseback riding result in a significantly higher surgery and hospitalization rate, as well as a significantly longer inpatient treatment compared to other sports injuries [4].

The most common injuries resulting from equestrian sports include head injuries, followed by injuries to the upper extremities and thoracic and lumbar spine. A large number of injuries, some of which are severe, with Injury Severity Scores (ISS) of up to 62 points, can be considered an indication that recreational riding can easily lead to life-threatening situations [2]. This epidemiological study was performed to investigate equestrian-associated injuries of the hand and wrist in a European study population based on data from a major hand trauma center in Northern Germany. We hypothesized that injuries caused by horseback riding are more severe and thus have a greater need for surgical treatment.

## Methods

Equestrian sports-related hand injuries treated between 2016 and 2021 at the Department of Plastic, Aesthetic, Hand and Reconstructive Surgery at Hannover Medical School, a level I trauma center, and a Federation of European Societies for Surgery of the Hand (FESSH)-accredited hand trauma center, were retrospectively reviewed. Patients with verified sports injuries of the hand were included, regardless of sex or age.

### Variables

A riding accident was defined as an accident resulting from handling a horse. In addition to falls from and with the horse, this included kicks and bites as well as accidents resulting from grooming or leading a horse by the rein or bridling a horse.

All included cases were reviewed for the following variables: age, sex, training status of the rider, wearing of protective gloves, horse size and sex, cause of injury, trauma mechanism, injury location, treatment type, hospitalization, and length of hospital stay.

### Data acquisition

Physician discharge letters and operation reports of the above-mentioned department were extracted from the Enterprise Clinical Research Data Warehouse (ECRDW) of the Hannover Medical School, which includes clinical routine data (such as diagnoses, laboratory findings, and operations). All collected data were exported from the ECRDW to Microsoft Excel spreadsheets. The medical reports of these cases were extracted for further processing. Finally, 39 patients with hand injuries and equestrian-related injury etiology were included in the analysis, as 7 were lost to follow-up. The loss of follow-up was due to some injuries being treated in an outpatient setting, leading to missed follow-up appointments. Consequently, we were unable to gather complete information on the injury mechanism and horse involvement.

### Statistical analysis

Statistical analyses were performed using IBM SPSS Statistics software (version 26.0; IBM, Armonk, New York, US). The results are given as mean ± SEM (standard.

error of mean) and range. For the analysis of nominal-scale variables, chi-square and Fisher’s exact tests were used. Statistical significance was defined as *P* < 0.05.

### Ethics, consent, and permissions

Ethical approval was waived by the local Ethics Committee in view of the retrospective nature of the study and all the procedures being performed were part of the routine care. The study was performed in accordance with the ethical standards of the institutional and national research committee and with the 1964 Helsinki declaration and its later amendments. Informed consent was not obtained as a study was done in an anonymized retrospective manner.

## Results

### Patients

Due to a loss-to-follow-up rate of 15.3% (*n* = 7) a total of 39 patients with equestrian-associated hand injury were analyzed. Overall, women were affected more often than men (female *n* = 35, 89%; male *n* = 4, 10%). The mean age was 35.1 ± 2.6 years (range 9–65).

Most of the riders (51%, *n* = 20) were hobby riders who had already achieved a riding badge for their participation in the sport. Overall, 41% (*n* = 16) of the riders did not have a badge and only 7.7% (*n* = 3) were professional riders. Wearing protective gloves was not common. Only 30.8% (*n* = 13) of the participants wore protective gloves when handling horses. Protective gloves were most frequently used by hobby riders with badges (40.0%, *n* = 8), followed by professional riders (33.3%, *n* = 1), and least frequently by hobby riders without badges (18.8%, *n* = 3).

To characterize horse sizes, we divided the horses and ponies based on the height at the withers measured from the ground. A horse or pony of any breed is called a pony if it is less than 1.47 m tall, whereas a horse is considered taller than 1.47 m. There were 53.8% (*n* = 21) horses and 46.2% (*n* = 18) ponies in the cohort. We observed that all professional riders (100%, *n* = 3) and 60% (*n* = 12) of riders with badges handled a horse, whereas the majority of hobby riders 62.5% (*n* = 10) without badges handled a pony. Most of the horses and ponies were female (59.0%, *n* = 23), followed by geldings (28.2%, *n* = 11), and stallions (12.8%, *n* = 5).

### Injuries

**Fig. 1:**
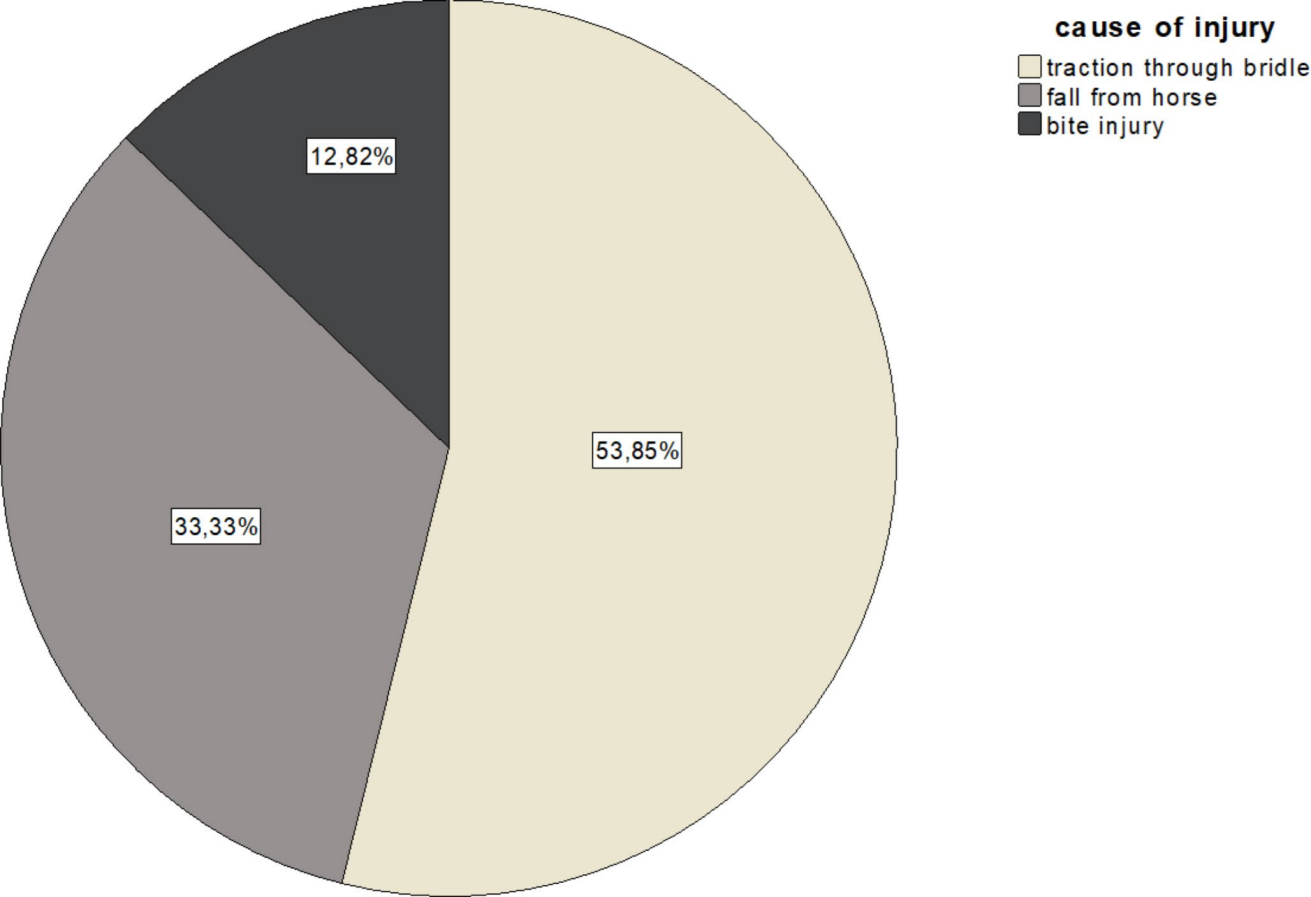
Most common causes of equestrian-associated injuries

The mechanism leading to most hand injuries was traction through a bridle (53%, *n* = 21), followed by falls from horses (33%, *n* = 13), and bite injury (12.82%, *n* = 5) (Fig. 1). Most injuries occurred at the phalanges (84.2%, *n* = 33), followed by the metacarpal bones (10.3%, *n* = 4) and the carpal bones (5.1%, *n* = 2).

**Fig. 2:**
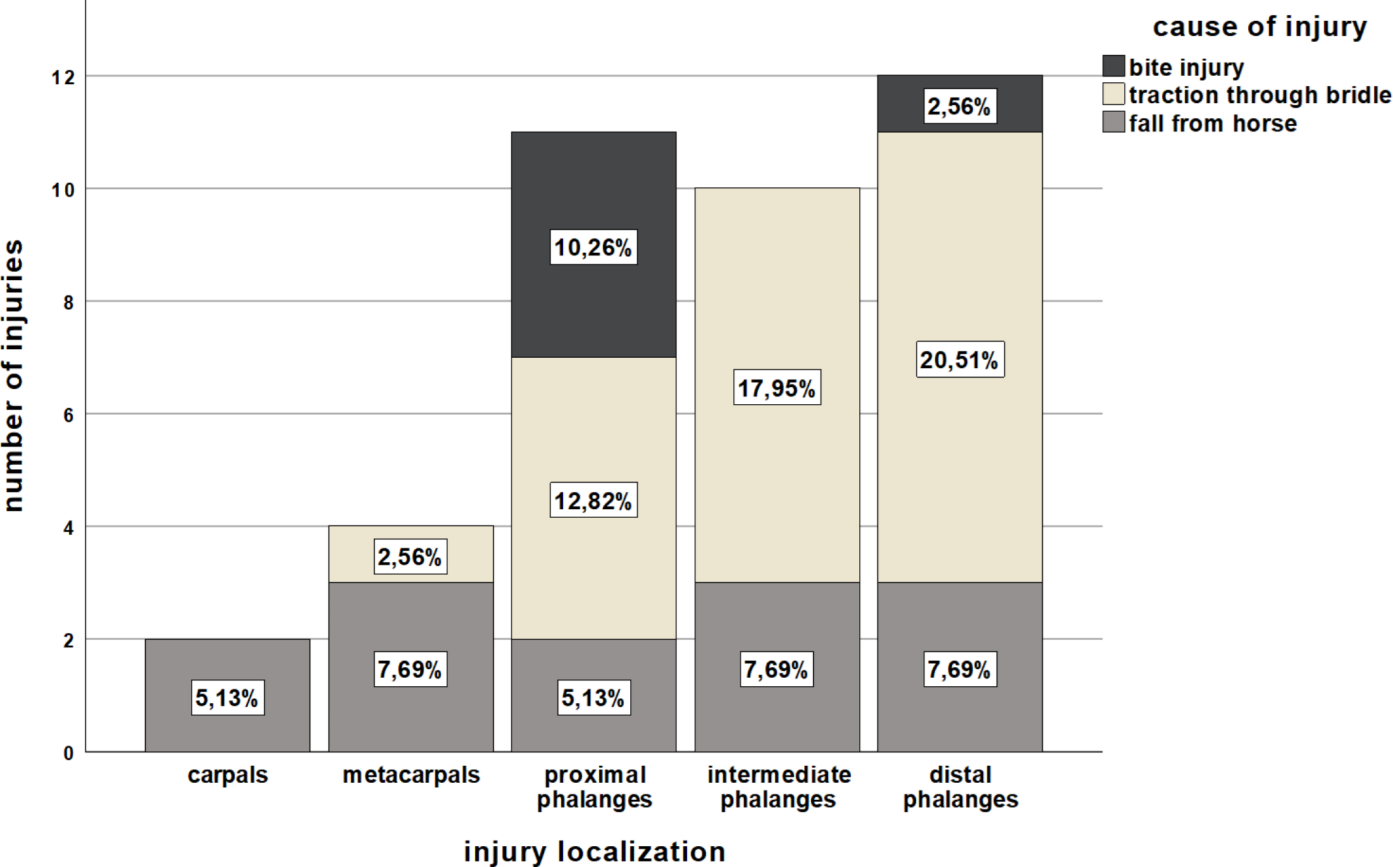
Location of injury stratified by cause

When the causes of injury were classified according to location, fall injuries appeared to be distributed approximately equally from the carpal bone to the distal phalanx, whereas traction and bite injuries occurred predominantly in the phalanges (Fig. 2).

**Fig. 3:**
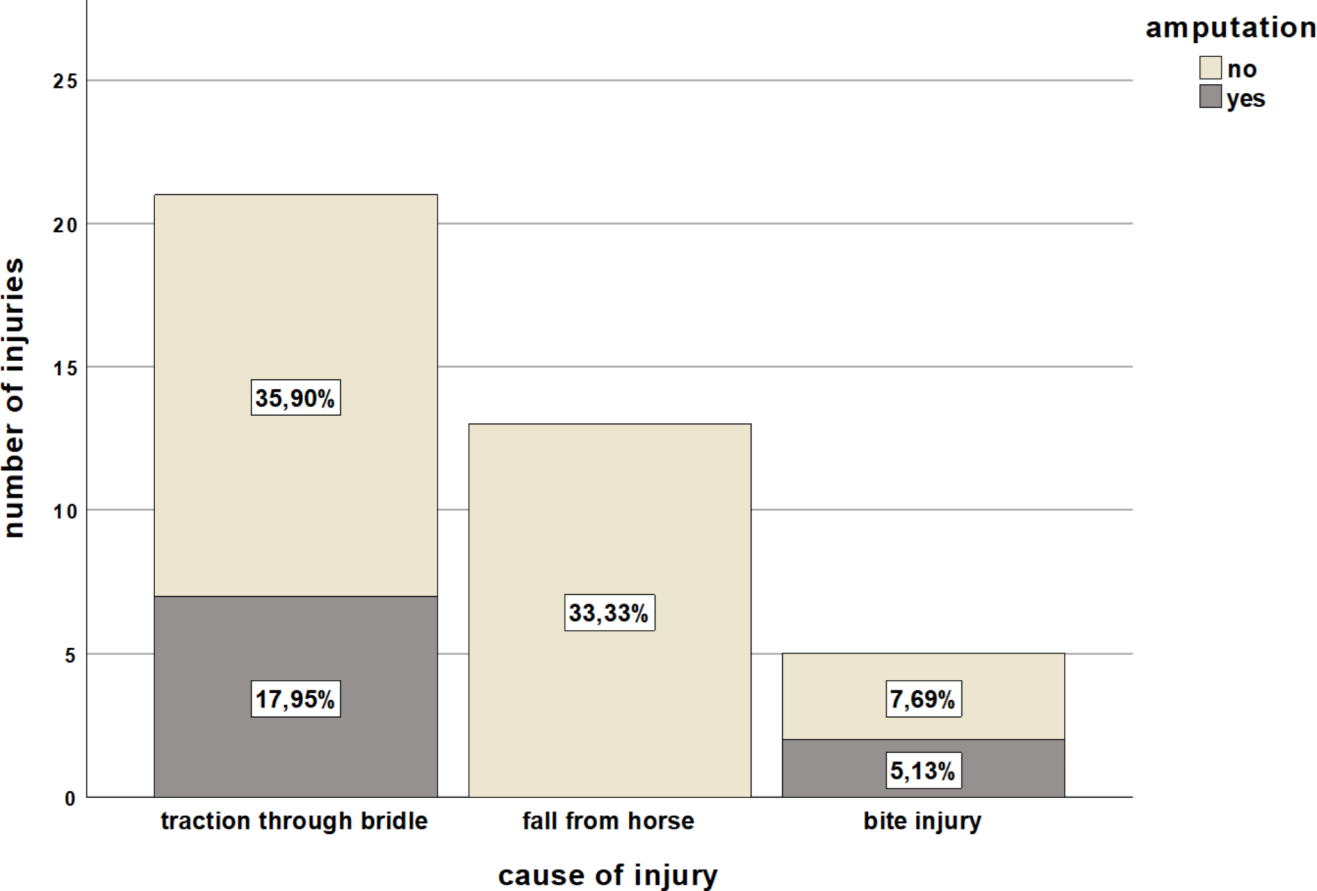
Overview of amputation injuries caused by traction through a bridle, fall from a horse, and bite injury

Among the injuries, we observed fractures in more than half of the patients (51.3%, *n* = 20), tendon injuries in 30.8% (*n* = 12), vascular injuries in 25.6% (*n* = 10), and nerve injuries in 30.8% (*n* = 12). Amputation injuries occurred in 23.1% (*n* = 9) of patients. Considering the proportion of amputations (Fig. 3) in terms of cause of injury, the highest rate was noted in bite injuries (40%, *n* = 2), and injuries caused by traction through a bridle (33.3%, *n* = 7). None of these occurred after a fall from a horse. Table 1 provides an overview of factors that may contribute to an amputation injury.

**Table 1:** Overview of factors that may contribute to an amputation injury

Factors		Amputation injury (%, *n* / all patients)	No amputation injury (%, *n* / all patients)	*P* value
Training status of the rider	hobby rider	25% (4/16)	75% (12/16)	0,61
	rider with badge	25% (5/20)	75% (15/20)	
	professional rider	0% (0/3)	100% (3/3)	
Wearing protective gloves	yes	0% (0/12)	100% (12/12)	**0**,036*
	no	30% (9/27)	0% (0/27)	
Horse size	horse	38,1% (8/21)	61,9% (13/21)	**0**,023*
	pony	5,6% (1/18)	94,4% (17/18)	
Horse sex	mare	34,8% (8/23)	65,2% (15/23)	0,106
	stallion	0% (0/5)	100%(5/5)	
	gelding	11,1% (1/11)	90,9% (10/11)	
Activity	horse care	30% (6/20)	70% (14/20)	**0**,03*
	lunging	50% (3/6)	50% (3/6)	
	riding	0% (0/13)	100% (13/13)	
Trauma mechanism	traction through bridle	33% (7/21)	66% (14/21)	0,05
	bite injury	40% (2/5)	60% (3/5)	
	fall from horse	0% (0/13)	100% (13/13)	

*A significant result was found for the wearing of protective gloves, horse size and activity

### Therapy

Surgical intervention was required in 79.5% (*n* = 31) of cases. The average inpatient stay was 1.41 ± 0.41 (range 0–13) days. This supports our hypothesis that horseback riding injuries are more severe and thus have a higher need for surgical intervention.

## Discussion

In this study, we investigated the circumstances, mechanisms, and location of horse-related hand injuries and the use of protective equipment in Northern Germany. We hypothesized that the most severe hand injuries were traction injuries caused by reins rather than by riding itself. The specific objectives were to estimate the effects of trauma mechanisms, rider demographic characteristics, riding experience, wearing protective equipment, and horse size and sex on the likelihood of amputation injury. The finding that most riding accidents were caused by reins and bridles confirmed our hypothesis.

### Sex distribution

Considering the sex distribution, 89% of the patients in our study were women. Compared with previous studies from Germany, a similar sex distribution was observed (72–94%) [5, 6]. Regarding the female majority in our study population, there are differences in equestrian-related activities between nations and countries. In the USA, a 10-year analysis of the National Trauma Database revealed a 50/50 distribution between men and women [7]. In contrast, women are predominant in equestrian sports in Europe. Two studies from the Netherlands and France found that 81% of individuals with horse-related injuries were female [8, 9]. Furthermore, a Swedish study found that up to 98% of individuals with injuries were women [10]. Therefore, our study population comprising 89% women is comparable to that in previous studies but makes it almost impossible to find sex differences due to the small subgroup of male patients.

In our study, only 7.7% of the participants were professional riders. Most riders (92.3%) were amateur riders, some of whom had earned badges as part of their equestrian training. In a retrospective study conducted in Berkshire, Whitlock et al. found a significantly higher percentage of professional riders with equestrian-related injuries (31.1%, *n* = 32) [11]. A different injury pattern was observed in the current study. Most jockeys sustained injuries to limbs, while the remainder sustained injuries mainly to the head and face. This could be because jockeys wear better head protection and since jockeys usually also fall on grass compared to amateur riders. Furthermore, jockey clubs have strict safety standards, and any jockey injured during a race is not allowed to start until declared fit to do so by a doctor.

### Injury mechanisms

Several studies from Europe have demonstrated that upper extremity injuries are the most common equestrian-associated injuries and that this could be due to a difference in personal protective equipment [6, 9, 12]. For risk prevention in horseback riding, we investigated the mechanisms leading to the aforementioned hand injuries. Thus, three categories were formed. The mechanism leading to the most hand injuries was traction through a bridle (53%), followed by falls from a horse (33%), and bite injuries (12.8%).

In 2013, Hessler et al. examined 189 injured riders treated at the BG Hospital in Berlin and Hamburg [13]. Overall, 72 (36.4%) riders fell from the horse, 16 (8.1%) had an accident due to a hoof strike, 10 (5.1%) fell with the horse, 3 (1.5%) collided with an obstacle during a ride, and 2 (1%) riders sustained horse-bite injuries. In 95 (48%) cases, the accident mechanism was unknown [13]. Compared to our study findings, the rate of fall injuries was similar; however, there was a significantly greater rate of bite and traction injuries. This discrepancy may be attributed to differences in study populations or reporting practices.

### Bite injuries

Among the bite injuries, our study found an amputation rate of 40%. Owing to its strong jaw and sharp teeth, a horse bite can cause serious injuries. Staszyk et al. studied the chewing movements of horses. These included opening, closing, and power strokes. Using a quartz crystal force sensor, chewing forces were recorded in 12 large horses (six females and six males, aged 5–27 years) on the second or third premolar of the mandible during chewing. The highest measured chewing force was 1758 N [14]. The biting force of horses is almost five times greater than that of humans (354 N) [15]. This shows that a horse exerts a great bite force, which frequently leads to serious injuries. In addition, horse bites can lead to polymicrobial infections due to colonization by a mixture of aerobic and anaerobic bacteria [16].

### Amputations

**Figs. 4, 5, 6:**
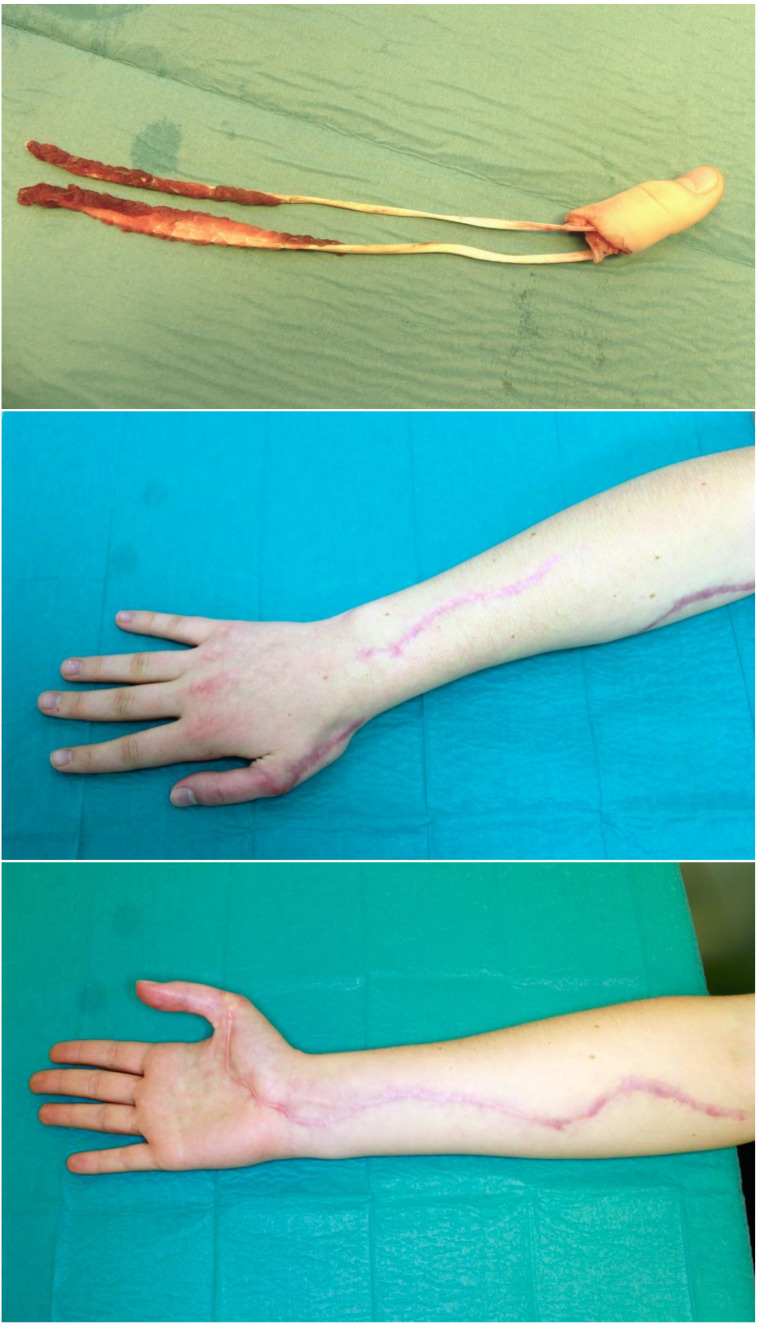
The images represent an 18-year-old female patient who was leading a horse in an indoor riding arena with a rope wrapped around her thumb. During an unexpected movement of the horse, a traumatic avulsion amputation occurred, resulting in an intra-articular multifragmentary basal phalanx fracture and long-distance avulsion of both the flexor and extensor tendons, as well as the neurovascular bundles. The complex replantation was successful, with a good functional outcome, including recovery of grip function

Avulsion amputation of the finger can be triggered by various causes including traffic accidents, industrial accidents, sports injuries, and household accidents. These are serious injuries in which the tissue, skin, muscle, tendon, or bone is torn from the hand by a sudden and traumatic force. This type of amputation presents a significant challenge to surgeons, as it can significantly affect the recovery of hand function and aesthetics, especially if it involves the thumb, which is responsible for around 20–25% of all hand function [17] (Figs. 4, 5 and 6). In our study, we found that amputations occurred in 23.1% of patients. The highest rates of amputations in terms of cause of injury were due to bite (40%) and traction (33.3%) injuries. The factors that significantly favored amputation were handling a large horse and not wearing protective gloves. Hoffmann et al. reviewed level I trauma center patients with horse-related injuries between 2019 and 2022 and found that in 95 patients with horse-related injuries, injuries to upper extremities accounted for 52.5% of injuries, followed by spinal and pelvic injuries (23.2%). Ten patients had their fingers tangled while holding the reins and sustained injuries to the upper extremities (*p* < 0.001). Three of them required amputation (30%) [6]. Abu-Zidan et al. reported a similar pattern of injuries in a study involving 213 horse-related injuries. In this population, seven patients (3%) became entangled in the reins and required hospitalization, but no injuries or treatments were discussed [18]. American rodeo riding is a special case, however, with a similar injury pattern. Morgan et al. reported three male patients with thumb injuries sustained during a rodeo-roping competition. The thumbs of two patients were amputated, while the third patient’s thumb was partially preserved. The mechanism of injury was identical in all three cases. The thumbs were caught between the rope and saddle horn when the rope slackened and subjected to shearing and crushing forces when the rope was placed under tension. These injuries illustrate the importance of keeping the thumb up and free of the rope to avoid entrapment between the rope and saddle horn [19].

### Epidemiology of horse-related hand injuries

Hand and wrist injuries account for up to 30% of all emergency department visits [20]. These injuries often lead to substantial pain, functional impairment, and reduced productivity. Severe cases, especially horse-riding related, can cause prolonged recovery times and pose a risk of long-term disability, which in turn results in significant financial burdens for both patients and healthcare providers [3, 4, 20]. Tamulevicius et al. recently reported a comprehensive 16-year analysis of hand injuries at our center. During the study period, 6,634 patients presented to our hand surgery emergency department, including 1,047 cases of wrist and hand fractures and 186 amputations [21]. According to this data, horse-related hand injuries account for approximately 1% of all emergency department visits. However, the rates of wrist and hand fractures and amputations are significantly higher, at 1.91% and 4.84%, respectively. Regarding the national and international relevance of finger and metacarpal fractures, it is important to note that these injuries have an incidence up to 1,483 per 100,000 inhabitants, making them the most common fractures of the upper extremity [22]. Given the high complication rates associated with surgical interventions (32–36%), adequate and early treatment is crucial [22]. This suggests that while horse-related injuries represent a small fraction of total emergency visits, they are disproportionately severe when they occur, emphasizing the need for targeted preventive measures and specialized care to address the high rate of serious outcomes associated with these injuries.

## Conclusion

In summary, injuries associated with horseback riding are more likely to occur while handling the horse than while riding and can result in severe avulsion amputations from pulling on the lunge or bridle lead, requiring complex microsurgical treatment. Injuries while leading a horse can be prevented by holding the reins, halter rope, or lead rope without wrapping the reins or rope around the fingers or thumb. In addition, wearing appropriate protective gloves should be considered for accident prevention, and it should be a mandatory part of equestrian training. Additionally, the use of self-opening panic hooks with overload protection can prevent excessive pulling.
